# Shear Stress: An Underrecognized Driver of Endothelial Inflammation in Acute Ischemic Stroke

**DOI:** 10.3390/cells15090772

**Published:** 2026-04-24

**Authors:** Yann L. Cordes, Huy Viet Dao, Nikolaos Zapantis, Vivian Vogt, Michael K. Schuhmann, Axel Haarmann

**Affiliations:** Department of Neurology, University Hospital Würzburg, 97080 Würzburg, Germanyvogt_v@ukw.de (V.V.)

**Keywords:** shear stress, thrombo-inflammation, brain endothelium, stroke, platelet activation, collateral flow, piezo1, syndecan-1, BBB, blood–brain barrier

## Abstract

Cerebral ischemic stroke is caused by impaired blood flow to the brain parenchyma due to acute vessel occlusion. Although current therapies focusing on rapid restoration of blood flow achieve high rates of recanalization, outcomes remain unfavorable in a significant proportion of patients. Part of this discrepancy is due to intravascular inflammation driven by thrombo-inflammatory mechanisms that add to cerebral tissue loss. Despite being an inevitable consequence of vessel occlusion, altered shear stress remains largely overlooked as a contributor to endothelial dysfunction in stroke. To directly assess the impact of disturbed flow on the endothelial phenotype, human brain endothelial cells were cultured under controlled flow conditions using an ibidi pump system and exposed to flow alternating in both magnitude and direction. Subsequently, the expression of key endothelial proteins, including Claudin-5, PECAM-1, CD62e and endoglin, was analyzed. We show here that the sequence of shear-stress modulation, recapitulating the hemodynamic conditions of large-vessel occlusion and subsequent reperfusion in stroke, is sufficient to cause an inflammatory phenotype in human brain endothelial cells. In addition, we demonstrate that platelet activation induces the mechanosensors Piezo1 and syndecan-1, sensitizing brain endothelial cells to shear-stress alterations characteristic of ischemic stroke. Targeting shear-stress-mediated inflammatory activation of the brain endothelium may therefore offer a complementary strategy in stroke therapy, particularly in large-vessel occlusion with abrupt flow changes.

## 1. Introduction

In ischemic stroke, cerebral blood flow has been extensively studied with regard to parenchymal perfusion. While recanalization is essential to favorable clinical outcomes, secondary complications such as edema and hemorrhagic transformation frequently occur and are partly driven by thrombo-inflammatory mechanisms [1,2]. However, the inflammatory consequences of altered shear stress at the endothelial interface during vascular occlusion and subsequent reperfusion, as well as the interaction between flow-derived mechanical signals and local inflammatory cues in human cerebral endothelium, remain poorly understood. Following large-vessel occlusion, pial collaterals rapidly supply the ischemic penumbra, partially maintaining tissue viability [3]. At the same time, collateral flow delivers blood to the affected vasculature, providing a reservoir of cells that can participate in localized platelet and granulocyte activation, potentially affecting endothelial barrier integrity [4,5,6]. Abrupt changes in blood flow or even flow reversal is likely to further disturb cerebrovascular homeostasis, directly activating cerebral endothelial cells via mechanosensitive ion channels such as Piezo1 and indirectly via the endothelial glycocalyx [7,8]. A key component of the latter is syndecan-1 (SDC-1), a transmembrane proteoglycan that links shear forces at the endothelial surface to the actin cytoskeleton [9,10]. A deeper understanding of how collateral flow and reperfusion-induced endothelial activation contribute to localized cerebrovascular inflammation may reveal complementary therapeutic targets aimed at limiting secondary infarct progression after ischemic stroke.

## 2. Materials and Methods

### 2.1. Cell Culture

Human brain microvascular endothelial cells (HBMECs; ACBRI 376, Cell Systems, Kirkland, WA, USA) were maintained at 37 °C in a humidified 5% CO_2_ atmosphere in gelatin-coated culture flasks. Cells were grown in Endothelial Cell Medium (ECM; Sciencell, Carlsbad, CA, USA) supplemented as recommended by the manufacturer and were used at passages below 6. For flow assays, cells were seeded on µ-Slides I 0.6 (ibidi, Gräfelfing, Germany) and incubated for 24 h until firm adhesion of the cells and subconfluence (1 × 10^6^ cells/mL per slide).

Slides were connected in series and exposed to flow for 24 h using an ibidi pump system at a shear stress of 6.2 dyn/cm^2^, representing a pragmatic compromise that falls within the physiological range of wall shear stress reported for a broad spectrum of cerebral microvessels, including small arterioles and capillaries [11]. For reverse low-flow conditions, shear stress was reduced to 1 dyn/cm^2^, and the slide orientation was inverted. Reflow was established by repositioning the slides to restore antegrade flow and applying a shear stress of 6.2 dyn/cm^2^.

### 2.2. Generation of Platelet Releasate

Whole blood from healthy donors was treated with EGTA (2 mM) and apyrase (0.2 U/mL) and allowed to rest for 30 min at room temperature (RT) to minimize spontaneous activation. Platelet-rich plasma was obtained by centrifugation (200× *g*, 10 min), diluted with citrate–glucose–saline buffer, and further cleared of residual blood cells by low-speed centrifugation (68× *g*, 10 min). Platelets were then pelleted (1500 × g), washed, and resuspended in calcium-containing Tyrode’s buffer. Activation was induced using sterile glass beads combined with vortexing and ultrasonication. After removal of debris by high-speed centrifugation (11,300× *g*, 10 min, RT), the supernatant (platelet releasate) was collected and stored at −80 °C until further use.

### 2.3. qRT-PCR

Total RNA was extracted using TRIzol reagent (Invitrogen, Waltham, MA, USA) following the manufacturer’s protocol. Complementary DNA (cDNA) was then generated using the TaqMan^®^ Reverse Transcription Kit (Applied Biosystems, Carlsbad, CA, USA). Quantitative real-time PCR (qRT-PCR) was performed in duplicates with TaqMan^®^ chemistry on a StepOne Plus system (Applied Biosystems, Carlsbad, CA, USA). The inventoried FAM-MGB TaqMan^®^ probes Hs00207230_m1 (Piezo1) (#4453320, Thermo Scientific, Waltham, MA, USA) and Hs04966523_m1 (Syndecan-1) (#4448892, Thermo Scientific, Waltham, MA, USA) were used for amplification. Gene expression levels were calculated relative to 18S rRNA (#4319413E, Applied Biosystems), which served as the endogenous control, as it was identified as the more stable reference gene under platelet releasate (Ptl-R) stimulation compared with GAPDH.

### 2.4. Western Blotting

For preparation of whole-cell protein lysates, cells were cultured on ibidi slides as described above. After stimulation under various shear-stress conditions, the culture medium was removed. Cells were then collected by applying ice-cold cell lysis reagent (Cellytic MT, Sigma-Aldrich, Burlington, MA, USA), supplemented with a protease and phosphatase inhibitor cocktail (cOmplete™ mini, #04693159001; PhosSTOP™, #04906837001; both from Roche, Basel, Switzerland). Cell suspensions were incubated with vigorous agitation for 30 min to ensure efficient lysis, followed by centrifugation at 18,200× *g* for 30 min at 4 °C. The clarified supernatants were collected and used for subsequent Western blot analysis. Primary antibodies were used against CLDN5 (1:500; 35-2500; Invitrogen, Waltham, MA, USA), PECAM-1 (1:1000; 3528; cell signaling, Danvers, MA, USA), endoglin (1:500; 3A9 #14606; cell signaling, Danvers, MA, USA), CD62e (1:350; 20894-1-AP; Proteintech, Rosemont, IL, USA), Piezo1 (1:500; #MA5-32876; Invitrogen, Waltham, MA, USA), syndecan-1 (1:1000; #52953; cell signaling, Danvers, MA, USA) and beta-actin (1:250,000; A5441; Sigma, St. Louis, MO, USA). Appropriate peroxidase-coupled secondary antibodies (donkey anti mouse IgG, #719039190, and donkey anti rabbit IgG, #711039192; both diluted to 1:10.000; both from Jackson ImmunoResearch Laboratories, West Grove, PA, USA) were employed with an enhanced chemoluminescence system (Perkin Elmer, Waltham, MA, USA).

## 3. Results

### 3.1. Altered Shear Stress Modulates Tight Junction and Adhesion Molecule Expression in Human Brain Endothelial Cells

**Figure 1:**
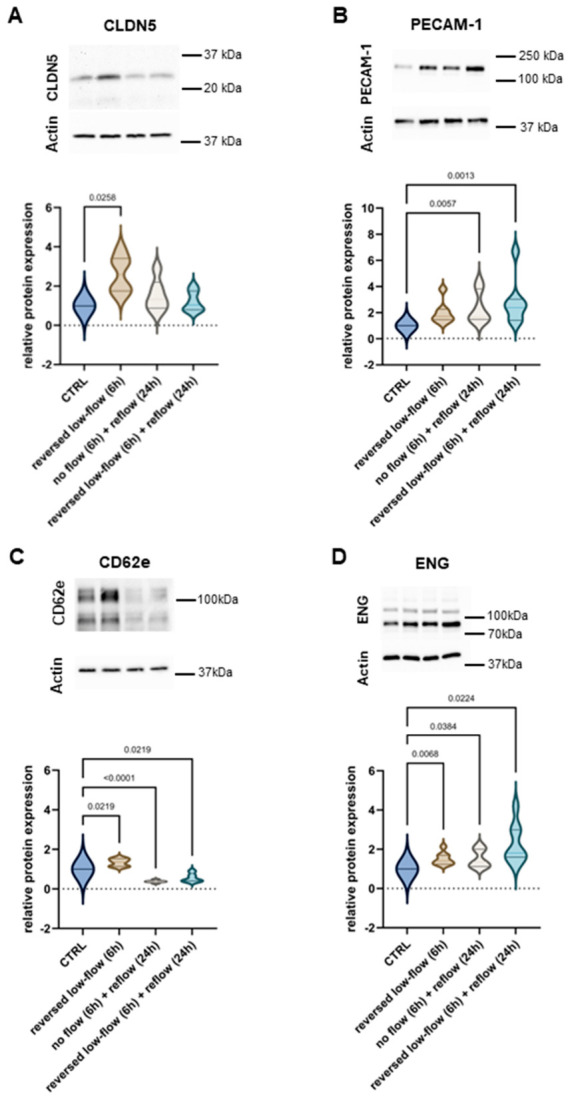
Distinct modulation of human brain endothelial protein expression in response to altered shear stress. (**A**) CLDN5 protein expression is upregulated upon exposure to reversed low-flow conditions. (**B**) PECAM-1 is significantly induced after stasis and low-flow with subsequent reperfusion. (**C**) CD62e showed opposing regulation increasing under reversed low flow and decreasing upon reperfusion following low flow or stasis. (**D**) ENG expression increased under all conditions of altered shear stress, with the most pronounced changes being observed under reversed low flow followed by reperfusion. Upper panels show representative Western blots. Violin plots represent *n* = 5–8 independent experiments. Different shear stress conditioned as indicated: (I) six hours of reversed low flow, mimicking pial collateral circulation; (II) six hours of reversed low flow followed by antegrade reflow, resembling recanalization in the presence of good collateral circulation; or (III) six hours of stasis followed by antegrade reflow. Statistical analysis was performed using one-way repeated-measures ANOVA for datasets that passed the Shapiro–Wilk normality test. For the PECAM-1 dataset, which did not meet normality assumptions, the nonparametric Friedman test was applied.

To investigate the direct effects of altered shear stress on the human brain endothelium, HBMECs were cultured under continuous-flow conditions for 24 h. Following this period, the endothelial monolayers were exposed to one of three flow conditions: (I) six hours of reversed low flow, mimicking pial collateral circulation; (II) six hours of reversed low flow followed by antegrade reflow, resembling recanalization in the presence of good collateral circulation; (III) six hours of stasis followed by antegrade reflow. Western blot analysis revealed robust induction of the tight junction protein claudin-5 (CLDN5) under reversed low-flow conditions, with a 2.6-fold increase (95% CI: 1.6–3.6; *p* = 0.0258) (Figure 1A). CLDN5 is the most abundant claudin at the blood–brain barrier and is essential to maintain its characteristic low paracellular permeability [12]. The induction of CLDN5 was transient and declined within 24 h of reperfusion. Platelet Endothelial Cell Adhesion Molecule-1 (PECAM-1), which strengthens cell–cell contact via homophilic interactions between adjacent endothelial cells, also plays a key role in leukocyte diapedesis [13,14]. PECAM-1 showed a trend toward increased expression under reversed low-flow conditions (1.99-fold; 95% CI: 1.2–2.8), although this was not statistically significant. Following reperfusion, both after preceding stasis and reversed low-flow, PECAM-1 was significantly upregulated, with a 2.4-fold increase (95% CI: 1.3–3.5; *p* = 0.0057) and a 2.9-fold increase (95% CI: 1.2–4.5; *p* = 0.0013), respectively (Figure 1B). E-selectin (CD62e), is an adhesion molecule involved in leukocyte rolling and plays a key role in the recruitment of neutrophils and monocytes via interaction with P-selectin glycoprotein ligand-1 (PSGL-1) [15,16]. In line with the formerly reported accumulation of neutrophils in the ischemic vasculature [5], CD62e was upregulated within 6 h of reversed low-flow conditions (1.3-fold increase, 95% CI: 1.1–1.6; *p* = 0.0219; Figure 1C).

Interestingly, CD62e expression was markedly reduced in the reperfused vasculature following both stasis (2.7-fold decrease, 95% CI: 2.2–3.4; *p* < 0.0001) and reversed low-flow treatment (1.8-fold decrease, 95% CI: 1.2–3.7; *p* = 0.0219). Endoglin (ENG), a TGF-β co-receptor, has recently been described to be induced in human brain endothelium under hypoxia, shed upon reoxygenation and characterized as a biomarker indicative of reperfusion following cerebral large-vessel occlusion [17,18]. Correspondingly, ENG expression was significantly upregulated after reperfusion in conditions preceded by stasis (1.52-fold increase, 95% CI: 1.1–1.9; *p* = 0.0384) and reversed low-flow (2.3-fold increase, 95% CI: 1.4–3.1; *p* = 0.0224) (Figure 1D).

### 3.2. Platelet Releasate Induces Piezo1 and SDC-1 Levels in Brain Endothelium

**Figure 2:**
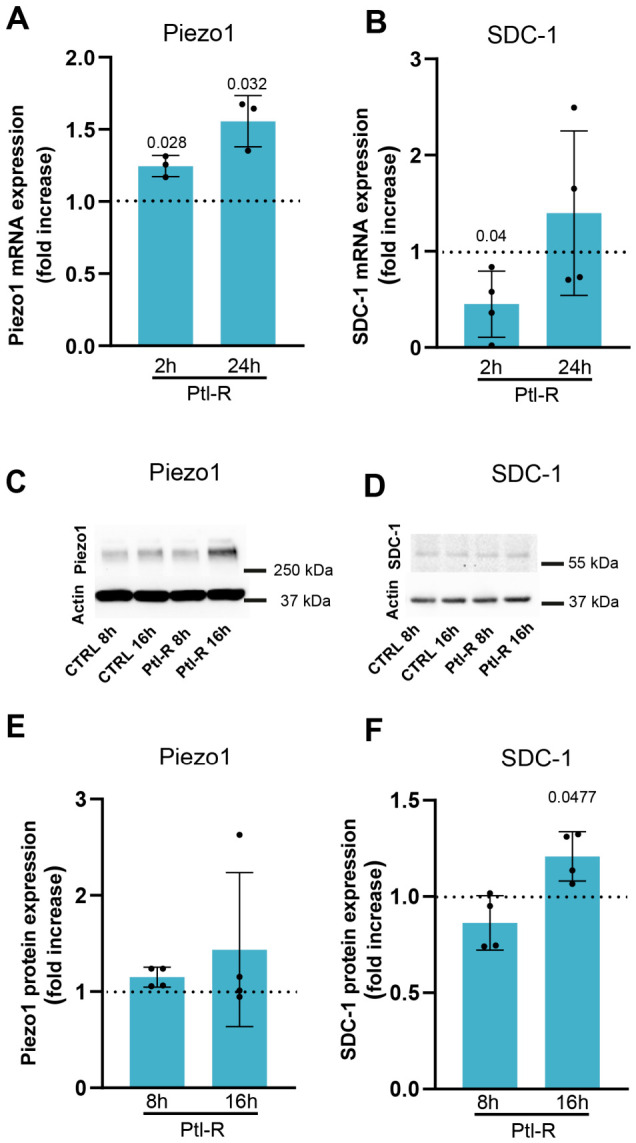
Modulation of mechanosensitive endothelial proteins by platelet activation. (**A**) Stimulation of HBMECs with Ptl-R for 2 or 24 h significantly induced Piezo1 mRNA transcription (*p* = 0.028 and 0.032, respectively, mean ± SD; paired *t*-test to corresponding control condition). (**B**) The levels of the glycocalyx component SDC-1 transiently decreased within the first 2 h of Ptl-R stimulation (*p* = 0.04, mean ± SD; paired *t*-test to corresponding control condition). (**C**) At the protein level, there was a trend toward higher Piezo1 expression after 16 h of Ptl-R stimulation; however, this did not reach statistical significance (see quantification in (**E**)). (**D**,**F**) Western blot analysis of SDC-1 showed increased protein expression after 16 h of Ptl-R stimulation (*p* = 0.0477; mean ± SD; paired *t*-test vs. corresponding control).

In stroke, the brain endothelium experiences hemodynamic alterations alongside thrombo-inflammatory activation, creating a complex environment in which mechanosensitive pathways may be modulated. While multiple cell types contribute to the inflammatory milieu, platelets release a particularly complex mixture of over 300 bioactive proteins from their α-granules upon activation [19]. These platelet-derived mediators accumulate within affected vessels and can be readily detected in the ischemic vasculature of patients undergoing mechanical thrombectomy for large-vessel occlusion stroke [4,6]. To assess whether Ptl-R modulates mechanosensitive protein expression in the human brain endothelium, HBMECs were treated with Ptl-R, after which Piezo1 and SDC-1 mRNA levels were quantified by qRT-PCR. Piezo1 expression was significantly increased after 2 h (1.25-fold increase, SD 0.07; *p* = 0.028) and 24 h (1.56-fold increase, SD 0.18; *p* = 0.032) (Figure 2A). At the protein level, we observed a trend toward upregulation of Piezo1 after 16 h of stimulation with Ptl-R. However, despite the significant changes observed at the mRNA level after 2 h, these protein level changes did not reach statistical significance (Figure 2C,E).

**Figure 3:**
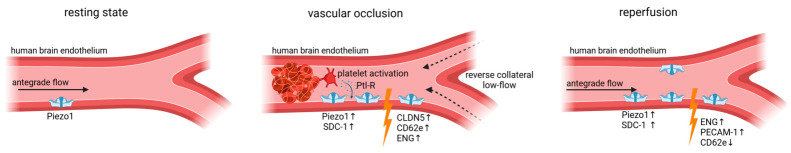
Scheme illustrating the intersection of shear-stress-mediated and thrombo-inflammatory mechanisms at the brain endothelium. Upon vessel occlusion, antegrade blood flow is disrupted in distal vessels. Pial collaterals, partially compensating the lack of antegrade perfusion, generate reverse collateral flow in the ischemic vasculature. At the same time, local platelet activation triggers endothelial inflammatory responses, leading to acute upregulation of Piezo1. This, in turn, modulates endothelial sensitivity to shear stress, affecting both the response to collateral low-flow and to reperfusion in case of recanalization. CLDN5, Claudin-5; CD62e, E-selectin; ENG, endoglin; PECAM-1, Platelet Endothelial Cell Adhesion Molecule-1; Ptl-R, platelet releasate. Created in Biorender. Axel Haarmann (2026).

In contrast, SDC-1 transcription showed a transient decrease after 2 h of stimulation before returning toward baseline levels at 24 h (Figure 2B). In Western blot analysis, SDC-1 protein levels were significantly increased at 16 h (Figure 2D,F). Together, these results suggest that localized platelet activation in the ischemic vasculature alters mechanosensitive protein expression during the acute phase of ischemic stroke, potentially priming human brain endothelial cells to respond more strongly to shear stress and influencing vascular function as well as stroke pathology (Figure 3).

## 4. Discussion

In acute ischemic stroke, shear-stress-mediated alterations and thrombo-inflammatory processes converge at the brain endothelial surface, potentially amplifying endothelial dysfunction and contributing to poor outcomes despite restored vessel patency. We show that even short-term changes in shear stress act as potent, distinct endothelial stimuli, shaping barrier integrity through modulation of intercellular junctions and regulating leukocyte–endothelial interactions via adhesion proteins. Notably, localized platelet activation appears to induce Piezo1 and syndecan-1 upregulation, potentially sensitizing endothelial cells to flow-dependent activation in the context of acute vessel occlusion.

As a mechanosensitive ion channel, Piezo1 is critical to maintaining endothelial barrier integrity, and its activation by disturbed flow or mechanical stress can disrupt adherens junctions and promote endothelial remodeling [20,21]. Inflammatory stimuli such as TNF-α have been shown to induce Piezo1 expression in endothelial cells in association with NF-κB pathway activation, supporting a mechanism by which inflammation sensitizes mechanotransduction responses in the vascular endothelium [22]. In contrast, SDC-1 is downregulated under the same conditions, consistent with prior reports of reduced mRNA expression following TNF-α, IL-1β, or LPS stimulation in endothelial cells [23,24].

Importantly, flow-mediated endothelial activation may represent a key component of the pathophysiology underlying inflammatory changes observed in hyperacute stroke: Upregulation of CD62e, a key receptor for PSGL-1, under conditions of reversed low-flow aligns with the accumulation of neutrophils in the occluded vessels via collateral circulation in patients with acute ischemic stroke [5]. Leukocyte accumulation, in turn, has been demonstrated to impair microvascular perfusion in animal models and human stroke, highlighting the tight interplay between hemodynamics and intravascular inflammation [25,26]. In addition, the flow-dependent increase in ENG expression supports our previous observation that soluble ENG levels reflect reperfusion in large-vessel occlusion stroke, correlating with infarct size and the rate of recanalization [17].

These findings highlight that in addition to restoring vessel patency, changes in blood flow critically influence endothelial activation, leukocyte recruitment, and barrier function. Our model shows that changes in shear stress alone can trigger endothelial activation, but it does not permit conclusions about the extent to which this mechanism contributes to the multifactorial pathophysiology of ischemic stroke in vivo. As an in vitro system using cultured endothelial cells, it does not recapitulate the cellular complexity of the neurovascular unit. Although the ibidi flow system enables control of shear-stress magnitude and direction, it represents only a simplified approximation of the hemodynamic conditions during large-vessel occlusion and reperfusion. Moreover, our analyses focus primarily on endothelial phenotypic markers. Therefore, functional validation of barrier integrity and mechanotransductive signaling remains limited.

## 5. Conclusions

Together, these findings show that altered shear stress alone induces distinct changes in tight junction and adhesion molecule expression in human brain endothelial cells. Ptl-R additionally modulates mechanosensitive endothelial proteins, including Piezo1 and SDC-1, at both the mRNA and protein levels. These results indicate coordinated endothelial responses to changes in flow and platelet-derived stimuli. A further understanding of how endothelial cells respond to changes in shear stress and flow direction may provide a mechanistic framework for therapies that not only restore perfusion but also limit inflammatory injury and improve functional outcomes in ischemic stroke.

## Data Availability

The original contributions presented in this study are included in the article material. Further inquiries can be directed to the corresponding author.

## Abbreviations

The following abbreviations are used in this manuscript:

HBMEC	Human Brain Microvascular Endothelial Cell
CD62e	E-Selectin
CLDN5	Claudin 5
PECAM-1	Platelet Endothelial Cell Adhesion Molecule-1
ENG	Endoglin
SDC-1	Syndecan-1
ANOVA	Analysis of Variance
Ptl-R	Platelet Releasate
PSGL-1	P-selectin Glycoprotein Ligand-1
